# A Systematic Review of the Cardiotoxic Effects of Targeted Therapies in Oncology

**DOI:** 10.7759/cureus.66258

**Published:** 2024-08-06

**Authors:** Wilhelmina N Hauwanga, Billy McBenedict, Emmanuel S Amadi, Taha K Dohadwala, Chukwuwike Johnny, Felix Asaju, Onyinye D Okafor, Abdulmalik Jimoh, Ada Andrea Oghenerukevwe Elumah, Okam V Onyinyinyechi, Dulci Petrus, Bruno Lima Pessôa

**Affiliations:** 1 Family Medicine, Federal University of the State of Rio de Janeiro, Rio de Janeiro, BRA; 2 Neurosurgery, Fluminense Federal University, Niterói, BRA; 3 Internal Medicine, Hallel Hospital Port Harcourt, Port Harcourt, NGA; 4 Medicine, David Tvildiani Medical University, Tbilisi, GEO; 5 Family Medicine, Lifepoint Medical Centre, Abuja, NGA; 6 Oncology, Missouri State University, Springfield, USA; 7 Internal Medicine, Mount Horeb Clinic and Dialysis Center, Warri, NGA; 8 Internal Medicine, Alimosho General Hospital, Lagos, NGA; 9 Family Health, Directorate of Special Programs, Ministry of Health and Social Services, Windhoek, NAM

**Keywords:** ejection fraction, cardioprotective, cancer, cardiotoxicity, chemotherapy

## Abstract

Cancer therapy advancements have improved survival rates but also introduced significant cardiotoxic risks. Cardiotoxicity, a critical adverse effect of cancer treatments such as doxorubicin, trastuzumab, and radiotherapy, poses substantial challenges. This systematic review synthesizes findings from studies on cardiotoxicity induced by cancer therapies, focusing on detection and management. Key predictors of chemotherapy-induced myocardial toxicity (CIMT) include advanced age, hypertension, hyperlipidemia, diabetes, and elevated N-terminal pro-B-type natriuretic peptide levels. Regular echocardiographic assessments, particularly of the left ventricular global longitudinal strain (LVGLS) and left ventricular ejection fraction (LVEF), are essential for early detection. The CardTox-Score, incorporating these risk factors, shows high sensitivity and specificity in predicting CIMT. Advanced imaging techniques and biomarkers play crucial roles in identifying at-risk patients before functional decline. Early biomarkers and imaging techniques such as LVGLS and LVEF are effective in diagnosing and managing cardiotoxicity, allowing timely interventions. Cardiology involvement in patient care significantly enhances adherence to cardiac monitoring guidelines and reduces cardiotoxicity risks. Management strategies emphasize regular cardiac monitoring, patient education, and the use of cardioprotective agents. A collaborative approach between cardiologists and oncologists is vital to assess cardiovascular risks, minimize vascular toxicity, and manage long-term adverse effects, ensuring the safety and efficacy of cancer therapies. This review underscores the importance of early detection and proactive management of cardiotoxicity in cancer patients to optimize treatment outcomes and improve quality of life.

## Introduction and background

Cancer comprises a bewildering assortment of diseases that kill 7.5 million people each year [1]. According to estimates from the National Cancer Institute, the number of cases has drastically increased in the last few decades. Neoplasms represent an important cause of morbidity and mortality in the world. Cancer was the second-leading cause of death in the United States overall and the leading cause among people younger than 85 years [2]. Cancer mortality in the United States declined by 33% from 1991 to 2021 due to reduced smoking, earlier detection, and advancements in targeted therapies and immunotherapy. Despite this, cancer incidence continues to rise for six of the top 10 cancers, including breast, prostate, uterine corpus, pancreas, oropharynx, liver (female), kidney, melanoma, colorectal, and cervical cancer in young adults [3]. Cancer disparities persist, particularly among women of African descent and Asian individuals, with the latter having the highest cancer incidence, mortality, and poverty rates [3].

Globally, cancer incidence is rising, a trend expected to continue due to population aging. However, advancements in cancer therapy have reduced mortality rates in many countries, resulting in a growing population of cancer survivors facing long-term complications of cancer therapy. Advancements in treatment modalities have improved cancer survival rates and cure possibilities. However, these treatments can cause significant adverse effects, including treatment-related cardiotoxicity, which may lead to a poorer prognosis than the cancer itself and impact the continuation of treatment [2]. Cardiotoxicity is a critical adverse effect associated with various cancer therapies, posing significant challenges in oncology. Cardiotoxicity, which refers to cardiac damage and dysfunction, is a severe cause of comorbidity and mortality in cancer therapies associated with treatments such as doxorubicin (DOX), trastuzumab (TZ), sunitinib, imatinib, ponatinib, and radiotherapy [4]. Despite extensive research, the pathophysiology of cancer therapy-induced cardiotoxicity (CTIC) remains unclear, and there is an unmet clinical need to identify patients at risk of developing CTIC, especially heart failure (HF). Imaging techniques and biomarkers are essential to detect at-risk patients before cardiac functional decline becomes apparent. Additionally, more targeted therapeutic options are required, as current treatments are limited to standard HF medications such as angiotensin-converting enzyme inhibitors and beta-blockers [4].

Cancer chemotherapy antibiotics can induce cardiotoxic effects at any stage of treatment, categorized into four types: acute, subacute, chronic, and late-onset cardiotoxicity, each with distinct clinical presentations and prognoses [5]. Acute cardiotoxicity, occurring in 0.4%-41% of patients, manifests as repolarization disturbances, reduced QRS complex voltage, sinus tachycardia, and QT interval prolongation, typically resolving spontaneously. Subacute cardiotoxicity is rare, emerging days to weeks post-anthracycline (AC) treatment, presenting as pericarditis-myocarditis syndrome, with a higher incidence of pericarditis in patients treated with daunorubicin than DOX. Chronic cardiotoxicity, occurring in 0.4%-23% of treated patients, results from repeated exposure to AC antibiotics, leading to severe congestive HF weeks to months after chemotherapy, primarily affecting the left ventricle. Late-onset cardiotoxicity, diagnosed years post-chemotherapy, affects both children and adults, even at low DOX doses (<480 mg/m²), presenting as congestive HF, arrhythmias, and conduction abnormalities, although rarely causing sudden death [5].

The emergence of chemotherapy-induced myocardial toxicity (CIMT) is a critical issue in cancer treatment, impacting patient outcomes and survival rates. A study involving 225 patients treated with non-AC myocardiotoxic anticancer agents found that 48.8% (n = 110) received alkylating agents, 47.3% (n = 106) were treated with monoclonal antibodies such as TZ, and 9.3% (n = 21) received immune checkpoint inhibitors (ICIs) such as ipilimumab [6]. All participants underwent echocardiography before, during, and after their anticancer therapy. CIMT developed in 11.1% (25 patients), marked by a significant reduction in left ventricular ejection fraction (LVEF) from 57.2% to 39.2% and a decrease in left ventricular global longitudinal strain (LVGLS) from -18.5% to -9.1% (p < 0.0001) [6]. Key predictors of CIMT included being over 60 years old, having arterial hypertension, hyperlipidemia, diabetes, and elevated N-terminal pro-B-type natriuretic peptide levels (>400 pg/mL). The newly established CardTox-Score, which incorporates these risk factors, showed high sensitivity (100%) and specificity (84.2%) in predicting CIMT. For effective monitoring and management, regular echocardiographic assessments, especially of LVGLS strain and LVEF, are essential, along with timely interventions such as dose adjustments or the initiation of cardioprotective therapies to reduce the risk of severe cardiac dysfunction [6].

Protecting cardiac function remains a significant challenge for the pharmaceutical industry, regulatory authorities, and physicians managing adverse reactions to anticancer agents. Evaluating patients exposed to these drugs, analyzing associated risks, preventing and mitigating cardiac injury, monitoring cardiac function during and after treatment, and addressing chemotherapy-related cardiotoxicity have together generated extensive knowledge and data known as cardio-oncology [7]. This systematic review aimed to provide a comprehensive synthesis of information from studies reporting cardiotoxicity induced by cancer treatments, focusing on the incidence, predictive markers, and management strategies of cardiotoxicity associated with various cancer therapies. The review included both early and late-onset cardiac adverse events in diverse patient populations, aiming to identify key biomarkers, monitoring protocols, and imaging techniques that can enhance early detection, guide timely interventions, and improve overall cardiac outcomes for cancer patients.

## Review

Materials and methods

This systematic review adhered to the principles outlined in the Preferred Reporting Items for Systematic Reviews and Meta-Analyses (PRISMA) guidelines for the organization and reporting of its results [8]. An electronic search was performed across multiple research databases, including PubMed, Embase, and Scopus (Table 1). All databases were accessed on June 13, 2024, and subsequently, a search was performed.

**Table 1:** Summary of the search strategy from the various databases

Database	Search Strategy	Filters Used
PubMed	(cardiotoxicity(Title/Abstract)) AND ((cancer therapy(Title/Abstract)) OR (cancer therapies(Title/Abstract)) OR (cancer drugs(Title/Abstract))	Humans only, English language, exclude preprints, filter years 2020-2024
Embase	(cardiotoxicity:ab,ti AND 'cancer therapy':ab,ti OR 'cancer therapies':ab,ti OR 'cancer drugs':ab,ti) AND ((controlled clinical trial)/lim OR (randomized controlled trial)/lim)	Humans only, English language, filter years 2020-2024
Scopus	(TITLE-ABS-KEY (cardiotoxicity) AND TITLE-ABS-KEY ("cancer therapy") OR TITLE-ABS-KEY ("cancer therapies") OR TITLE-ABS-KEY ("cancer drugs"))	Humans only, English language, filter years 2020-2024

Inclusion criteria encompassed studies reporting cardiotoxicity from cancer therapy in adult humans (18 years and older), focusing on targeted therapies, immunotherapies, and newer chemotherapeutic agents. Eligible studies included primary research published in English from 2020 to 2024, such as randomized clinical trials, cohort studies, case-control studies, cross-sectional studies, and case series (>10 cases). The review sought studies on cardiotoxicity from cancer therapy that also report on management. Only peer-reviewed journal articles in English were included. Exclusion criteria involved animal studies, pediatric patients, studies on pre-existing cardiovascular disease without assessing or reporting cardiotoxicity attributable to cancer therapies, non-peer-reviewed articles, conference abstracts (due to limited data), editorials, opinion pieces, commentaries, letters to the editor, and reviews.

Results

**Figure 1:**
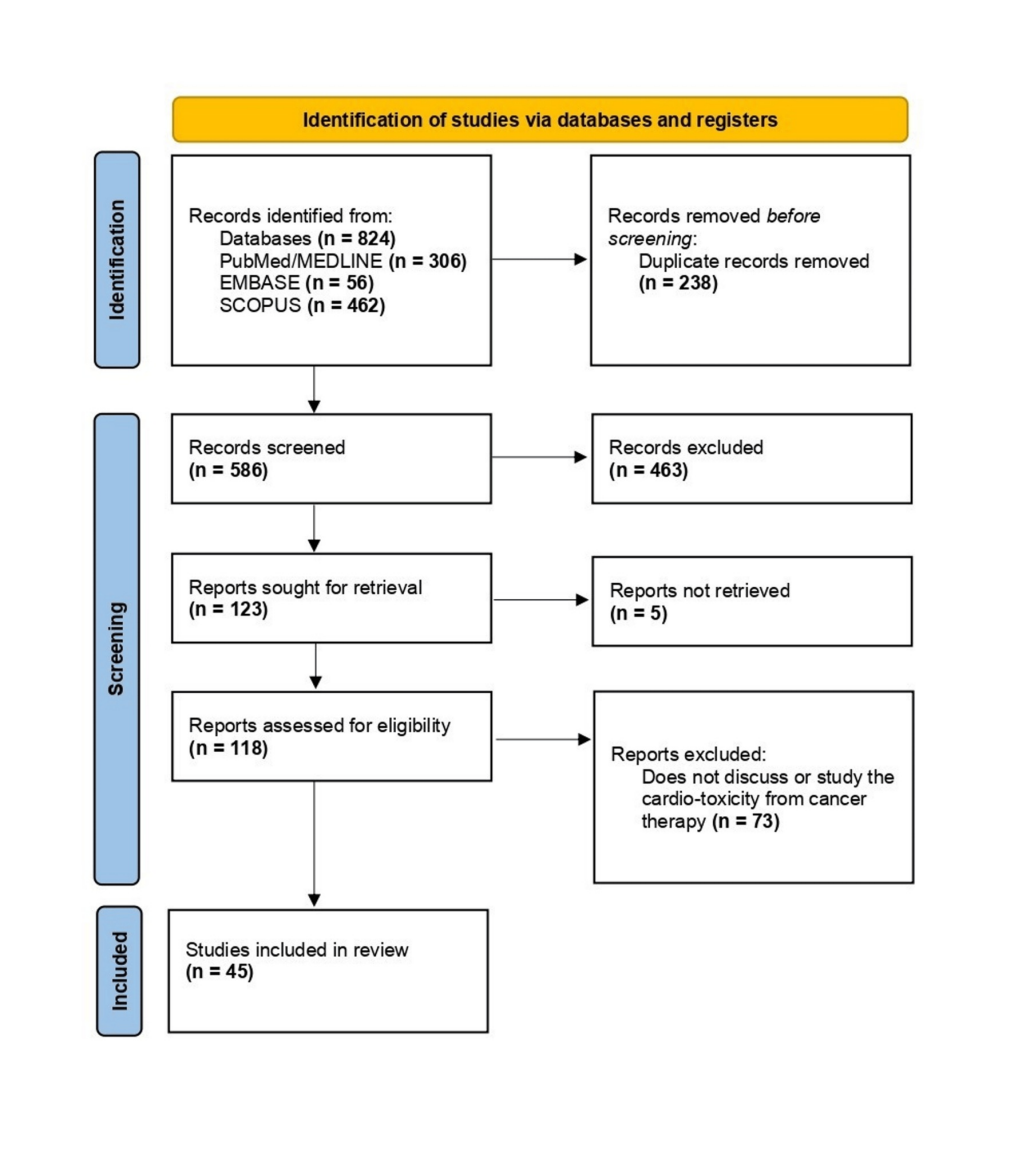
Preferred Reporting Items for Systematic Reviews and Meta-Analyses (PRISMA) flow diagram indicating the steps taken to filter the articles for this review

Through our search strategy, we identified a total of 824 articles (Figure 1), including 306 from PubMed/Medline, 56 from Embase, and 462 from Scopus. We applied filters based on the inclusion and exclusion criteria and then transferred the articles to an MS Excel sheet (Microsoft Corporation, Redmond, Washington). After manually removing 238 duplicates, 586 articles remained. These were further scrutinized based on titles and abstracts, leading to the exclusion of 463 articles, leaving 123 for further consideration. We were unable to retrieve the full texts for five articles, resulting in 118 papers eligible for assessment. Following a thorough full-text review, 73 papers were excluded, culminating in 45 articles included in the final review (Table 2). Data screening was independently conducted by two review authors, with a third reviewer consulted in cases of disagreement. Importantly, no automated tools were utilized in this process.

**Table 2:** Studies that were used to synthesize this review, with their respective demographics and key results CRT = chemoradiation therapy; NSCLC = non-small cell lung cancer; CAEs = cardiac adverse events; hs-cTnT = high-sensitivity cardiac troponin-T; SUV = standardized uptake values; RV = right ventricular; CTRCD = cancer therapy-related cardiac dysfunction; NT-proBNP = N-terminal pro-B-type natriuretic peptide; GLS = global longitudinal strain; c-LVEF = left ventricular ejection fraction by contrast‐enhanced echocardiography; ECG = electrocardiogram; AI = artificial intelligence; MRI = magnetic resonance imaging; LVEF = left ventricular ejection fraction; EF = ejection fraction; DOX = doxorubicin; ARI = activation recovery interval; STE = speckle tracking echocardiography; CMR = cardiovascular magnetic resonance; hiPSC-CMs = human-induced pluripotent stem cell-derived cardiomyocytes; ATP = adenosine triphosphate; LVGLS = left ventricular global longitudinal strain; ICIs = immune checkpoint inhibitors; CV = cardiovascular; LVSD = left ventricular systolic dysfunction; NSVT = non-sustained ventricular tachycardia; BTK = bruton tyrosine kinase; TKI = tyrosine kinase inhibitor; BNP = B-type natriuretic peptide; GDMT = guideline-directed medical therapy; AC = anthracycline; TZ = trastuzumab; PALS = peak atrial longitudinal strain; 5-FU = 5-fluorouracil; HER2 = human epidermal growth factor receptor 2; eBC = early breast cancer; ARBs = angiotensin II receptor blockers; ACE = angiotensin-converting enzyme; MACCEs = major adverse cardiac and cerebrovascular events; nCRT = neoadjuvant chemoradiation; AF = atrial fibrillation; PLD = pegylated liposomal doxorubicin; LV = left ventricular; CPT = cardioprotective therapy; LVESD = left ventricle end systolic diameter; ACS = acute coronary syndrome; PTX = paclitaxel; hsCRP = high-sensitivity C-reactive protein; TNF-a = tumor necrosis factor alpha; CK-MB = creatine-kinase (total CK), MB fraction; GCS = global circumferential strain; HF = heart failure; LGE = late gadolinium enhancement

Author	Type of Study	Key Findings
​Xu et al. [9]	The study included 225 patients, with 53% men and 47% women, and a median age of 66 years.	Cardiotoxicity from CRT for NSCLC is significant, with 11% experiencing severe CAEs; hs-cTnT levels effectively predict risk, suggesting routine monitoring to improve patient outcomes.
Kim et al. [10]	121 women, aged 27 to 80 years (mean 54).	Cardiotoxicity in breast cancer therapy with AC or TZ affects 12% of patients; elevated SUV in RV wall detected by F-fluorodeoxyglucose Positron Emission Tomography/Computed Tomography scans can predict and manage this risk.
Demissei et al. [11]	The study included 323 breast cancer patients (median age 48 years, mixed genders) treated with various cancer therapies​.	In breast cancer patients treated with AC and TZ, 14.2% experienced CTRCD; elevated hs-cTnT and NT-proBNP levels were strong predictors, highlighting the importance of biomarker monitoring.
Liu et al. [12]	The study included 111 breast cancer patients (average age 48.39 years, primarily female) and 77 control individuals​​.	In breast cancer patients treated with AC and targeted therapy, GLS at three months predicts late-onset CTRCD, enhanced by combining GLS with c-LVEF, emphasizing early, regular monitoring for timely interventions.
Yagi et al. [13]	The study included 1138 patients (average age 57.1 years, 47.8% male).	In AC-treated patients, AI models using baseline ECG effectively predict CTRCD, with high AI-CTRCD scores indicating significantly increased risk, enhancing 2-year prediction accuracy and guiding timely interventions.
​Kar et al. [14]	Of the 39 eligible adult female breast cancer patients, 32 participated in the study, with a mean age of 50.3 years.	In breast cancer patients treated with AC and TZ, GLS measured by MRI significantly worsened post-therapy, predicting CTRCD risk independently of LVEF; early GLS assessment and regular monitoring are crucial.
Upshaw et al. [15]	The study included 362 breast cancer participants with a median age of 49 years, predominantly Caucasian females (70%)​.	In breast cancer patients treated with DOX and TZ, 80% developed diastolic dysfunction within three years, evidenced by significant changes in echocardiographic measures; routine monitoring is essential for early detection and cardioprotection.
Jacobs et al. [16]	The study included 989 female breast cancer patients with a mean age of 64.89 years.	In breast cancer patients, AC therapy led to EF decline in 2.2% of cases over 9.8 years; the AI-ECG model effectively detected EF reduction, recommending it for early cardiotoxicity screening.
Koop et al. [17]	The study included 12 female breast cancer patients aged 42 to 73 years, with a median age of 59 years.​	In breast cancer patients with CTRCD, AC, and TZ, therapy significantly reduced LVEF and quality of life, causing fatigue, anxiety, and HF symptoms; regular cardiac assessments and multidisciplinary care are essential.
Kinoshita et al. [18]	The study included 85 patients with malignant lymphoma, comprising 57% males, with a mean age of 65 years​.	In patients with late-onset DOX-induced CTRCD, ARI prolongation significantly correlated with decreased LVEF; ARI monitoring via ECG can predict cardiotoxicity, enabling timely cardiac protection strategies.
Chang et al. [19]	125 patients (mean age not specified).	In cancer therapy with AC, patients developing cardiotoxicity showed LVEF reduction from 69.2% to 63.8% post-epirubicin; early LV longitudinal strain on the subendocardium reduction predicted cardiotoxicity, suggesting regular STE for early intervention.
Barac et al. [20]	The study included 11,732 women aged 24-64 with newly diagnosed breast cancer.	Baseline cardiac imaging is crucial for managing cardiotoxicity in early-stage breast cancer patients on AC or TZ treatments, with 79-81% adherence; however, only 16% of non-AC patients received imaging, necessitating improved monitoring for high-risk groups.
Kersten et al. [21]	The study included 34 female breast cancer patients with a mean age of 50.2 years.​	AC-based breast cancer therapies significantly reduce LVEF and RVEF, with CMR showing reductions from 65.2% to 61.1% and LV radial strain; native T1 and T2 times indicated myocardial inflammation, highlighting the need for regular imaging and cardioprotective strategies.
​Sang et al. [22]	The study incorporated 33,418 virtual patients.	DOX and TZ significantly increase systolic dysfunction risk; DOX dose-dependently, and TZ 0.31-2.7%, rising to 0.15-10% combined. hiPSC-CMs showed decreased contractile force and ATP levels. Early detection and cardioprotective strategies are crucial.
Chen et al. [23]	The study involved 33 breast cancer patients, aged 51.36 ± 9.77.	Two-dimensional-STE is more sensitive than traditional echocardiography for detecting early LV dysfunction from AC in breast cancer patients, with significant LVGLS deterioration after two cycles, enabling timely intervention.
​Toribio-García et al. [24]	195 elderly cancer patients (76.9% male, 23.1% female) with a mean age of 75 years.	Cardiotoxicity from ICIs in elderly cancer patients occurs in 1.54% of cases, primarily as myocarditis and arrhythmias; regular cardiac monitoring and early symptom detection are crucial for effective management.
​Shibata et al. [25]	486 patients, with a mean age of 63.8 years, 53.5% female.	Cardiotoxicity affects 16-38% of cancer patients, with CV events most frequent in multiple myeloma (38.1%); risk prediction tools and collaborative CV monitoring can mitigate long-term adverse effects.
​Glen et al. [26]	40 women, aged 62 ± 10 years.	In breast cancer survivors treated with TZ and AC, 25% experienced long-term LVSD; comprehensive CV risk assessments and regular monitoring are essential for managing elevated NT-proBNP, hs-cTnT, and lipid levels.
Wei et al. [27]	193 patients (75% male), with an average age of 76.9 years.	In patients with hematologic malignancies, ibrutinib is linked to higher rates of AF (44%) and NSVT (43%) than non-BTK TKIs and non-TKI therapies, necessitating regular CV assessments and cardiologist involvement.
Wang et al. [28]	500 patients (408 men, 92 women), mean age 60.98 years, treated for lung and esophageal cancer.	In 500 cancer patients, LVEF decreased significantly (68.51% to 66.23%), Cardiac troponin-T increased (7.42 to 14.54 ng/L), and extracellular volume rose (47.14% to 52.64%) after 12 months.​
Abboud et al. [29]	67 patients, 67.2% were female with a mean age of 69 years.​	Cardiotoxicity from oral oncolytics is common, with 46.2% experiencing HF; monitoring with echocardiograms, troponins, and BNP, alongside multidisciplinary management and GDMT, is essential for effective intervention.
Gąsior et al. [30]	155 women with breast cancer, aged 54.8 ± 9.8 years​.	Cardiotoxicity, defined as a >10% decrease in LVEF below 50%, occurred in 14.3% of breast cancer patients treated with AC and TZ, with the highest incidence (22%) in the AC + TZ group. Regular echocardiographic evaluations and cardioprotective medications are recommended for early detection and management.
​Di Lisi et al. [31]	169 women with breast cancer, aged 55 ± 10.8 years​.	In breast cancer patients treated with AC and TZ, cardiotoxicity was indicated by significant reductions in GLS and PALS despite stable LVEF; regular echocardiographic assessments using these measures are crucial for early detection and management.
Hammond et al. [32]	30 cancer patients undergoing 5-FU chemotherapy (ages 62.0 ± 12.9) and 32 controls, matched by age and sex​.	5-FU-based cancer therapy can cause cardiotoxicity, with 1-20% symptomatic incidence and over 50% experiencing asymptomatic ST segment changes due to microvascular dysfunction. Regular CV assessments, patient education, and cardioprotective agents are recommended to mitigate risks.
Posch et al. [33]	185 women with HER2+ eBC, with a median age of 55 years.	In HER2 + eBC, TZ therapy led to cardiotoxicity in 10%, predicted by a ≥5% LVEF decline. Regular LVEF monitoring every three months is crucial for early detection and management.
Lombardi et al. [34]	129 colorectal cancer patients (51 females, 78 males) with a median age of 69 years​.	In colorectal cancer patients, 15.5% experienced fluoropyrimidine-induced cardiotoxicity, with symptoms like dyspnea and chest pain. Regular ECG and biomarker monitoring, along with baseline cardiological assessments, are essential for managing risks and completing treatment safely.
​Buck et al. [35]​	33 ibrutinib-treated cancer patients (30% women), with a mean age of 65 years.​	Ibrutinib treatment in cancer patients shows high cardiotoxicity, with 54.8% developing LGE fibrosis and significant increases in native T1 and max-T2 values. Regular CMR, ECGs, and biomarker assessments are crucial for early detection and management of myocardial injury.
Lu et al. [36]	169 socioeconomically marginalized breast cancer patients (75% racial/ethnic minorities), with a median age of 51 years​.	In breast cancer patients, cardiotoxicity incidence was 21% at one year with TZ, 3.9% with AC; 59% had treatment interruptions, highlighting the need for close cardiac monitoring.
Valente et al. [37]	112 patients (55 in Unit A, 57 in Unit B), all female, with a median age of 53 years.	In cancer patients treated with TZ, 21.9% developed HF; regular LVEF assessments and antihypertensive medications like ARBs and ACE inhibitors are crucial for managing cardiotoxicity and improving outcomes.
​Chang et al. [38]	48,258 patients (50.18%) were female; the mean age was 66.93 years.	In NSCLC patients treated with TKIs, there is an increased risk of MACCEs, including HF, myocardial infarction, and stroke, but a lower risk of all-cause death; regular CV monitoring and early interventions are essential.
​Beukema et al. [39]	57 patients, with a median age of 54 years and a range of 27–77 years. All patients were female.	Cardiotoxicity from nCRT in esophageal cancer survivors includes higher rates of myocardial fibrosis and AF, necessitating advanced cardiac imaging and biomarker monitoring to detect and manage early cardiac toxicity.
Hu et al. [40]	The study analyzed 1,471 breast cancer patients (661 PLD, 810 epirubicin), median age 48.9 (22.5–73.3) years, 56.3% under 50.	Cardiotoxicity is a significant concern in breast cancer therapy, with PLD showing lower cardiotoxicity than epirubicin and better LVEF and ECG outcomes. Regular cardiac monitoring and cardioprotective agents are essential for management.
Lee et al. [41]	A total of 95 patients, aged 51 ± 14 years, including 41 males and 54 females, participated in the study.	Combining ICIs with DOX in sarcoma therapy leads to greater LVEF reduction and higher CTRCD incidence; regular echocardiography and serum troponin-T monitoring are essential for early detection and management.
Carpenter et al. [42]	32 female patients with breast cancer, aged 35-69 years.	The use of Doxil in preoperative chemotherapy for locally advanced breast cancer significantly reduces cardiotoxicity while maintaining efficacy, with no cardiotoxicity observed and 29% achieving pathological complete response. Regular cardiac function and blood pressure monitoring are essential for managing potential late-onset cardiotoxicity.
​Saijo et al. [43]	The study involved 87 patients (63% female) aged 58 years on average (range not specified)​.	Cardiotoxicity from DOX is significant, with LVEF and GLS decreasing post-treatment; basal longitudinal strain predicts CTRCD, necessitating regular echocardiographic monitoring for early detection and management.
​Thavendiranathan et al. [44]	307 patients, 94% female, aged 54 ± 12 years​.	In the SUCCOUR study, GLS-guided CPT significantly reduced CTRCD incidence in AC-treated patients compared to an EF-guided approach, with 5.8% versus 13.7% developing CTRCD, respectively; regular echocardiographic monitoring is essential.
Laufer-perl et al. [45]	The study included 103 female breast cancer patients, aged 35-69 years.	In breast cancer patients treated with TZ and pertuzumab, 5% developed CTRCD, with 10% experiencing significant LVEF reduction. Lower GLS and higher LVESD at baseline were predictors; routine GLS and echocardiographic follow-ups are essential for early detection and management.
​Esteban-Fernández et al. [46]	113 patients, 82.3% female, aged 49.2 ± 12.1 years​.	In a study of 113 patients, 37.5% developed CTRCD with a mean LVEF of 39.4±9.2%; 54.8% recovered with cardiac-specific treatment. Early diagnosis and regular LVEF monitoring, along with ACE inhibitors and beta-blockers, are crucial for improving prognosis and treatment continuity.
​Mrotzek et al. [47]	153 patients, 59% female, aged 49.2 ± 12.1 years.	In cancer patients treated with DOX and ICIs, 66% presented with ACS, with 1-year mortality at 46% versus 8% in non-cancer patients. Regular monitoring of cardiac biomarkers and guideline-based management, including dual antiplatelet therapy, is essential.
Micheletti et al. [48]	120 female breast cancer patients, aged 30-77 years.	In a study of 120 breast cancer patients, DOX, PTX, and TZ treatments significantly increased hsCRP, TNF-a, and triglyceride levels, indicating cardiotoxicity. Regular monitoring of markers like CK-MB and hsCRP, along with adherence to CV guidelines, is essential for early intervention.
​Demissei et al. [49]	1,047 female breast cancer patients, aged 54 years (median)​.	In breast cancer patients treated with TZ, cardiology involvement improved guideline-adherent cardiac monitoring (76.4% vs. 60.1%) and biomarker use (27.8% vs. 13.8%), and better-managed CV risk factors, reducing systolic blood pressure, particularly in hypertensive.
Singh et al. [50]	30 cancer patients (mean age 43.73) and 15 controls (mean age 42.76).	In cancer patients undergoing chemotherapy and/or radiotherapy, STE detected significant reductions in GLS and GCS over six months despite preserved LVEF, highlighting STE's sensitivity for early myocardial dysfunction detection. Regular STE assessments and timely cardioprotective interventions are recommended.
Lambert et al. [51]	60 participants (mean age: 49.8±11.6 years), 30 were women.	In breast cancer patients, those with CTRCD had greater temporal changes in LV function, with GLS changes of 1.9% versus 0.7% and CMR-LVEF changes of 6.6% versus 2.7%. Regular echocardiographic and CMR imaging are recommended for early detection and management of cardiotoxicity.
Hinrichs et al. [52]	485 cancer patients, the average age was 62 years, with a gender distribution of 51% female and 49% male​.	Cardiotoxicity from cancer therapy, leading to reduced LVEF, can result from various treatments. Despite elevated NT-proBNP levels, 43% with LVEF below 50% showed no NT-proBNP increase. Comprehensive monitoring includes biomarkers, clinical exams, ECG, and echocardiography for early myocardial dysfunction detection and timely intervention.
Dolladille et al. [53]	Most patients were men (78.9%) with a median age of 70 years​.	Cardiotoxicity from ICIs includes late CAEs, with a median onset of 304 days; 73.7% experienced LVSD, and 47.4% developed HF. Continuous cardiac monitoring beyond 90 days and management with beta-blockers, ACE inhibitors, and ivabradine are essential.

Analysis of Study Quality/Bias

The quality of the 46 articles was assessed using the JBI Critical Appraisal Tools (Appendix Table 3). The purpose of critical appraisal (assessment of risk of bias) is to evaluate the methodological quality of a study and determine how effectively it has minimized bias in its design, conduct, and analysis. Bias refers to systematic errors that can affect the validity of conclusions in quantitative studies. In systematic reviews, critical appraisal aims to identify potential biases in the included studies. All 46 articles included in this systematic review have been critically analyzed. All studies included in the analysis focused on a clearly defined issue regarding cardiac toxicity from cancer therapy. Despite differences between the included studies, they have all employed various methods to minimize biases. Each study recruited participants in an acceptable manner, clearly stating the inclusion criteria and participants' demographic data (Appendix Table 3).

Discussion

Cardiotoxicity from cancer therapy is a significant concern that necessitates careful monitoring and management. In a study involving 486 patients with hematologic malignancies and breast cancer, cardiovascular (CV) adverse events were reported in 16%-38% of patients, with specific occurrences in 24.5% of leukemia, 15.8% of malignant lymphoma, 38.1% of multiple myeloma, and 18.0% of breast cancer cases [25]. The Heart Failure Association and International Cardio-Oncology Society risk assessment tool effectively predicted high/very high-risk patients' likelihood of experiencing CV events, which were found to be higher in these groups, while CV deaths accounted for 0.8% of the cases during follow-up (a follow-up period of ≤30 days) [25]. Various anticancer drugs and their associated cardiotoxicity were examined, including ACs, which caused high incidences of heart failure and left ventricular systolic dysfunction (LVSD) in patients with leukemia, malignant lymphoma, and breast cancer. HER2-targeted therapies, used in breast cancer, increased the risk of heart failure and cardiomyopathy. Immunomodulatory drugs (iMiDs) and proteasome inhibitors, used in multiple myeloma, presented elevated risks of CV events, including thromboembolic events. Additionally, checkpoint inhibitors and targeted therapies, used across various cancers, showed emerging evidence of CV side effects such as myocarditis and pericarditis [25]. The study underscores the importance of implementing CV risk stratification and monitoring strategies to mitigate the impact of cardiotoxicity, highlighting the need for a collaborative approach between cardiologists and oncologists to enhance the safety and efficacy of cancer therapies.

Chemoradiation

Cardiotoxicity from cancer therapy, particularly chemoradiation therapy (CRT) for non-small cell lung cancer (NSCLC), is a significant complication that negatively impacts survival and quality of life. In a study involving 225 patients (median follow-up of 26.2 months) undergoing concurrent platinum and taxane-doublet chemotherapy with thoracic radiation therapy (60-74 Gy), 11% experienced grade ≥3 cardiac adverse events (CAEs) at a median of nine months post-therapy [9]. Platinum compounds (e.g., cisplatin) and taxanes (e.g., paclitaxel) are linked to cardiotoxic effects such as arrhythmias, myocardial infarction, and HF. High-sensitivity cardiac troponin-T (hs-cTnT) levels were found to be reliable biomarkers for early detection of CAEs, with pretreatment levels higher in men, older patients (≥64 years), and those with pre-existing heart conditions. The hs-cTnT levels increased significantly during CRT and correlated with mean heart dose and heart volumes receiving 5-55 Gy [9]. Elevated hs-cTnT levels before (≥10 ng/L) and during CRT (increase of ≥5 ng/L) were associated with increased risks of severe CAEs and mortality. Routine monitoring of hs-cTnT can facilitate early identification of high-risk patients, allowing for timely intervention and therapy modifications to mitigate cardiotoxicity. This proactive approach to monitoring and managing cardiotoxicity is crucial for improving patient outcomes ​[9]​.

A prospective cross-sectional pilot study with a follow-up period of 5-15 years after curative resection revealed that patients treated with neoadjuvant chemoradiation (nCRT) (combination of carboplatin and paclitaxel in the nCRT regimen) exhibited a higher incidence of myocardial fibrosis, with linear late gadolinium enhancement (LGE) observed in four out of 18 irradiated patients versus one out of 20 non-irradiated patients (p = 0.13)​ [39]​. Additionally, atrial fibrillation (AF) was more prevalent in the nCRT group (six vs. two, p = 0.07). Patients with AF received higher radiation doses to the atria, resulting in a lower ejection fraction (EF) (51.5% vs. 59.4%, p = 0.04) and worse performance on the six-minute walking test (64.8% vs. 74.7% of predicted, p = 0.10) [39]. Monitoring strategies emphasized the use of advanced cardiac imaging techniques, such as cardiac MRI and echocardiography, to detect subclinical cardiac toxicity​ [39]​. Management included close monitoring of biomarkers such as N-terminal pro-B-type natriuretic peptide (NT-proBNP) and high-sensitivity troponin-T, along with the use of cardioprotective medications when necessary. Early identification and intervention are critical for mitigating the long-term cardiac effects of nCRT in esophageal cancer patients [39].

Anthracycline or Trastuzumab

In cancer therapy, particularly with AC or TZ for breast cancer, cardiotoxicity remains a critical concern. A study of 121 breast cancer patients undergoing these treatments found that 12% exhibited cardiotoxicity post-therapy [10]. Over a follow-up period from January 2009 to December 2015, 121 patients were evaluated. Cardiotoxicity, defined as an absolute LVEF value below 50% or a decrease greater than 20% from baseline, was observed in 12% of patients. Patients with cardiotoxicity had significantly higher standardized uptake values (SUV) in the right ventricular (RV) wall (2.4 ± 1.1 vs. 1.6 ± 0.7) and an increased change in SUV (ΔSUV) (0.7 ± 1.1 vs. 0.1 ± 0.7) compared to non-cardiotoxic patients [10]. Visual and quantitative analyses using F-fluorodeoxyglucose positron emission tomography (PET)/computed tomography (CT) scans revealed that the presence of RV wall uptake and elevated SUV values were significantly associated with cardiotoxicity. Monitoring these PET parameters can aid in early detection and management of cardiotoxicity, allowing timely interventions to modify treatment and mitigate adverse cardiac effects. This approach emphasizes the importance of incorporating advanced imaging techniques for proactive cardiotoxicity management in cancer patients​ [10].

In a cohort of 12 breast cancer patients with cancer therapy-related cardiac dysfunction (CTRCD), the participants reported overwhelming fatigue, anxiety, and reduced social participation. These patients experienced significant declines in their LVEF, with reductions from a mean of 62.7% to 53.8% post-therapy [17]. Moreover, 60% of participants developed HF symptoms within one year of completing cancer treatment. Chemotherapy regimens, including ACs (DOX) and cyclophosphamide, are associated with significant cardiotoxic risks, leading to conditions such as HF, myocardial infarction, and valvular disease. TZ, a targeted therapy for HER2-positive breast cancer, is known to cause HF and other cardiovascular dysfunctions, with cardiotoxic effects evident in several patients who received this therapy alongside chemotherapy [17]. Hormone therapy, used for hormone receptor-positive breast cancer, also contributed to cardiovascular issues, although its direct cardiotoxic effects are less pronounced compared to chemotherapy and targeted therapy. To monitor and manage cardiotoxicity, the study underscores the necessity of regular cardiac assessments, including LVEF and biomarkers such as hs-cTnT [17]. Effective management should involve a multidisciplinary approach with personalized care plans, including cardioprotective strategies and close coordination between oncologists and cardiologists, to mitigate long-term cardiovascular risks and improve the quality of life for these patients.

In a study involving 989 breast cancer patients, 22 patients (2.2%) developed a decline in EF to below 50% due to AC therapy over an average follow-up period of 9.8 years. The artificial intelligence (AI)-electrocardiogram (ECG) model demonstrated high diagnostic performance with an area under the curve (AUC) of 0.93 for detecting EF <50% and 0.94 for detecting EF ≤35% [16]. Patients with cardiotoxicity showed a significant decline in EF from 62.7 ± 5.1% to 53.8 ± 13.2% post-therapy. For monitoring and management, the study recommends using AI-ECG as a cost-effective, non-invasive screening tool to detect early signs of cardiotoxicity, enabling timely interventions and reducing the need for more costly cardiac imaging ​[16]​. Another study involving 323 breast cancer patients treated with these therapies found that 14.2% experienced CTRCD, with the highest incidence (39.1%) in the DOX + TZ group [11]. Key biomarkers, including hs-cTnT and NT-proBNP, showed early increases, particularly with AC-based regimens. Elevated hs-cTnT levels (>14 ng/L) at AC completion were linked to a twofold increase in CTRCD risk (hazard ratio (HR)=2.01) [11]. NT-proBNP increases were associated with a 56% higher CTRCD risk per doubling of the biomarker (HR=1.56). Monitoring these biomarkers can enhance early detection and management of cardiotoxicity, allowing timely interventions to reduce CV risk [11]​.

In a study of 362 breast cancer patients treated with DOX and TZ, diastolic dysfunction was evident with significant reductions in the mitral early-diastolic inflow peak velocity (E)/late mitral inflow peak velocity (A) ratio, lateral and septal early-diastolic myocardial velocity (e') velocities, and increases in the E/e' ratio, particularly in those receiving DOX or combined DOX and TZ [15]. Specifically, incident diastolic dysfunction developed in 60% of participants at one year, 70% by two years, and 80% by three years post-therapy [15]. The study highlights the importance of routine echocardiographic monitoring to assess diastolic function, using measures such as E/A ratio, e' velocities, and E/e' ratio, to detect early signs of cardiotoxicity and implement timely cardioprotective strategies to mitigate long-term cardiac risks [15]. A study reported late-onset chronic DOX-induced CTRCD; the prolongation of the activation recovery interval (ARI) after chemotherapy was notably observed only in the CTRCD group. ARI was prolonged from 258 ± 53 ms to 211 ± 28 ms (p = 0.03), correlating with a decrease in LVEF, from 45.1% in CTRCD patients compared to 69.6% in non-CTRCD patients (p < 0.001) [18]. The optimal cut-off point for ARI prolongation to predict CTRCD was 18 ms, with a sensitivity of 75% and specificity of 79% [18]. Monitoring ARI through ECGs may enable early detection of cardiotoxicity, facilitating timely interventions to protect cardiac function. Effective management includes regular cardiac monitoring, particularly using ARI measurements, and early therapeutic strategies to mitigate the cardiotoxic effects of DOX​ [18].

A study incorporating 33,418 virtual patients found that DOX-induced systolic dysfunction occurred dose-dependently, while TZ alone led to a 0.31%-2.7% incidence of dysfunction [22]. This incidence increased to 0.15%-10% when combined with DOX. Monitoring cardiotoxicity using human-induced pluripotent stem cell-derived cardiomyocytes revealed a 5.4-fold decrease in contractile force and a 3.2-fold decrease in adenosine triphosphate levels within 72 hours when DOX was followed by TZ [22]. In another study involving breast cancer survivors treated with TZ and AC, 25% showed LVSD years after therapy [26]. Among 40 women studied, 30% had elevated NT-proBNP levels, and 8% had elevated hs-cTnT. Additionally, 58% had high total cholesterol, and 43% had elevated triglycerides. Regular CV evaluations, including magnetic resonance imaging, showed that participants with LVSD had significantly larger indexed left ventricular (LV) volumes and worse global longitudinal strain (GLS) indices [26]​.

In a study involving 34 breast cancer patients who underwent cardiotoxic therapy with follow-up periods of six and 12 months. The patients primarily received ACs (91.2%), taxanes (97.1%), and cyclophosphamide (85.3%), with some receiving Her2-targeted therapies such as TZ (23.5%) [21]. Cardiovascular magnetic resonance (CMR) revealed significant reductions in LVEF from 65.2% at baseline to 61.2% at six months and 61.1% at 12 months (p = 0.016) [21]. RVEF also decreased significantly. Additionally, there was a significant reduction in LV radial strain from 29.7% to 25.8% at 12 months (p = 0.008) [21]. Parametric mapping showed transient increases in native T1 and T2 times, indicative of myocardial inflammation. Monitoring and management should focus on early detection through regular imaging and potentially integrating cardioprotective strategies to mitigate long-term cardiac dysfunction​ [21].

In a study of 155 women, cardiotoxicity (a decrease in LVEF of more than 10% to a value below 50%) was observed in 20 patients (14.3%) over a 12-month follow-up [30]. The AC + TZ group showed the highest incidence at 22%, compared to 13% overall. Significant changes in diastolic parameters, such as an increase in left atrial volume index, were particularly noted in the AC + TZ group. Monitoring and management strategies include regular echocardiographic evaluations every three months and the use of cardioprotective medications to detect and mitigate early signs of cardiac dysfunction​ [30]. In another study involving 169 women, GLS and peak atrial longitudinal strain (PALS) were used to detect early cardiac dysfunction [31]. While LVEF remained stable (60 ± 1.7% at baseline to 59 ± 3.4% at six months, p > 0.05), significant reductions were observed in GLS (from -20.7 ± 2.1% to -18.9 ± 2.4%, p < 0.0001) and PALS (average from 36 ± 8.9% to 28.7 ± 6.3%, p = 0.0002) [31]. A PALS reduction of over 20.8% was identified as a threshold for detecting patients at higher risk of asymptomatic mild cardiotoxicity. Management and monitoring strategies include regular echocardiographic assessments, incorporating GLS and PALS measurements to identify subclinical cardiotoxicity early and guide cardioprotective interventions [31]​.

In a study with TZ-based cancer therapy with a total of 185 women, 19 patients (10%) experienced cardiotoxicity, with a median pre-treatment LVEF of 64% [33]. A decrease in LVEF by ≥ 5% during treatment was a strong predictor of subsequent cardiotoxicity. The risk of cardiotoxicity was higher in patients with pre-treatment LVEF < 60% and in those with early LVEF decline, highlighting the importance of regular LVEF monitoring every three months during therapy [33]. Management strategies should include dynamic assessment of LVEF trajectories to identify high-risk patients and adjust treatment plans accordingly ​[33]​.

In a study involving 169 women, the cumulative incidence of cardiotoxicity was 21% at one year and 25% at three years for those treated with TZ, while for AC-treated patients, it was 3.9% and 5.9%, respectively. Among patients experiencing cardiotoxicity, 59% had interruptions in their cancer treatment, with 38% unable to continue [36]​. Management involved medications such as angiotensin-converting enzyme (ACE) inhibitors, angiotensin II receptor blockers (ARBs), and beta-blockers. Notably, 81% of TZ-related cardiotoxicity cases resolved within a year, compared to none for AC. Close monitoring and management are crucial, involving baseline and periodic cardiac assessments, particularly echocardiograms, to detect early signs of cardiotoxicity and adjust treatment protocols accordingly​ [36]​. Regarding cardiotoxicity from TZ, a study found that 21.9% of patients developed HF, with symptoms such as dyspnea (13%), increased blood pressure (13%), and fatigue (11%) [37]. Hypertension was a prevalent comorbidity (53%), followed by smoking (33%) and diabetes mellitus (22%). Monitoring strategies include regular assessment of LVEF, where a reduction below 53% or a 10% decrease from baseline was indicative of cardiotoxicity [37]. Management involved the use of antihypertensive medications, primarily angiotensin receptor blockers (26%) and ACE inhibitors (20%), which showed cardioprotective effects. These findings underscore the importance of early detection and tailored therapeutic interventions to mitigate the cardiotoxic impact of cancer treatments and enhance patient outcomes​ [37]​.

In a study comparing pegylated liposomal doxorubicin (PLD) and epirubicin for stage I-III breast cancer, the results showed no significant difference in overall survival and disease-free survival between the two groups [40]​. However, PLD demonstrated lower cardiotoxicity, with better LVEF and ECG outcomes (p < 0.05)​ [40]​. This suggests that PLD could be a preferable option for patients, particularly those with pre-existing cardiac conditions. Effective monitoring and management strategies are essential, including regular cardiac function assessment using echocardiography and ECG. The use of cardioprotective agents, such as ACE inhibitors, ARBs, and beta-blockers, has shown potential benefits in mitigating cardiotoxic effects, although more research is needed to establish definitive guidelines ​[40]​​.

Combining ICIs with DOX in cancer therapy may cause more harm in comparison to a single therapy of either. A study evaluated 95 patients with sarcoma, with 22 receiving DOX and ICIs and 73 receiving only DOX [41]. At six months, the Dox-ICIs group showed a greater reduction in LVEF (55% vs. 59% in the DOX group) and a higher incidence of CTRCD (38.1% vs. 17.4%, p = 0.042) [41]. Additionally, serum troponin-T levels were significantly higher in the Dox-ICIs group (53.3 pg/mL vs. 27.5 pg/mL, p = 0.023) [41]. Monitoring strategies should include regular echocardiography, particularly LVGLS, and serum troponin-T levels to detect early cardiac dysfunction. Management involves discontinuing cardiotoxic therapy upon detection of CTRCD and initiating HF treatments, which can lead to the recovery of cardiac function [41].

The use of liposome-encapsulated DOX in preoperative chemotherapy for locally advanced breast cancer shows significantly reduced cardiotoxicity while maintaining efficacy. In a study involving 32 patients, no cardiotoxicity or grade 4 or 5 toxicities were observed [42]. The most frequent toxicities were rash (17/32) and palmar-plantar erythrodysesthesia (23/32), with only one case of grade 3 palmar-plantar erythrodysesthesia, mucositis, and proteinuria, and seven cases of grade 3 hypertension [42]. Importantly, all patients exhibited at least a 30% reduction in tumor size, and 29% achieved pathological complete response at operation [42]. Long-term follow-up (median of 87 months) indicated that 22 patients remained free of recurrence, and 27 were alive​ [42]​.

In a study assessing cardiac dysfunction, LVEF and GLS significantly decreased after AC administration, with LVEF dropping from 65% ± 4% to 63% ± 4% (p = 0.003) and GLS from 23.2% ± 2.6% to 22.2% ± 2.4% (p = 0.005) ​[43]. Worse basal longitudinal strain (LS) was strongly associated with the development of CTRCD. Among patients undergoing further follow-ups, 13% developed CTRCD, with basal-LS decrease being a significant predictor (HR = 0.60, 95% CI = 0.43 to 0.83, p = 0.002) ​[43]. Early detection and management are crucial, involving regular echocardiographic assessments, particularly monitoring changes in basal-LS and GLS, to initiate cardioprotective treatments promptly and mitigate progression to severe cardiac dysfunction ​[43]​. Another study found that patients who developed cardiotoxicity experienced a significant reduction in LVEF from 69.2% to 63.8% after three cycles of epirubicin (p = 0.005) [19]. Moreover, LV longitudinal strain on the subendocardium was significantly reduced early in the treatment, predicting cardiotoxicity with an odds ratio of 2.14 (p = 0.005) [19]. This layer-specific strain measure was more sensitive than conventional echocardiographic parameters. Effective monitoring includes regular cardiac evaluations using layer-specific speckle tracking echocardiography (STE) to detect early myocardial dysfunction, allowing timely intervention to prevent further cardiac damage​ [19]​.

Cardiotoxicity from cancer therapy with AC and TZ is a notable risk that impacts treatment outcomes and patient survival. In a study of 113 patients, the incidence of CTRCD was 37.5%, with a mean LVEF of 39.4 ± 9.2% at diagnosis ​[46]. Cardiac-specific treatment was initiated in 66.4% of patients, resulting in a 54.8% recovery rate of LVEF. Predictors for LVEF recovery included higher LVEF at CTRCD diagnosis, shorter time from chemotherapy initiation to CTRCD diagnosis, and younger age ​[46]. Mortality was associated with lower LVEF at diagnosis and TZ treatment. Monitoring and management strategies emphasized early diagnosis through regular echocardiographic assessments, particularly measuring LVEF and implementing cardiac-specific treatments such as ACE inhibitors and beta-blockers. This approach helps improve prognosis and allows the continuation of cancer treatment, thereby reducing the risk of treatment withdrawal due to cardiotoxicity ​[46]​. Similarly, in a study of 103 patients from the Israel Cardio-Oncology Registry, 5% developed CTRCD, with an additional 10% experiencing a reduction in LVEF of 5% or more [45]​. Baseline echocardiographic parameters significantly associated with CTRCD development included lower GLS (-18 ± 3% vs. -21 ± 2%, p = 0.016) and higher left ventricle end systolic diameter (35 ± 6 mm vs. 25 ± 4 mm, p < 0.001) [45]​. Patients treated with TZ and pertuzumab were more likely to develop CTRCD (80% vs. 18%, p = 0.001 for TZ and 80% vs. 14%, p < 0.001 for pertuzumab) [45]​. For monitoring and management, the study supports the routine use of GLS and regular echocardiographic follow-ups to detect early signs of cardiotoxicity, enabling timely initiation of cardioprotective therapy (CPT) to prevent severe cardiac dysfunction​ [45]​.

Cardiotoxicity from DOX, paclitaxel (PTX), and TZ was reported in a study of 120 breast cancer patients. DOX treatment increased high-sensitivity C-reactive protein (hsCRP) to 4.80 ± 1.23mg/dL (p = 0.0005), tumor necrosis factor alpha (TNF-a) to 42.31 ± 17.96 pg/mL (p = 0.01), and triglycerides to 187.6 ± 25.06 (p = 0.0231) [48]. PTX and TZ also led to significant increases in creatine-kinase (total CK), MB fraction (CK-MB), hsCRP, cholesterol, and TNF-a levels. Monitoring involves assessing markers such as CK-MB and hsCRP, alongside advanced imaging techniques. Management includes adhering to CV guidelines and monitoring pre-existing conditions to mitigate the risks of cardiotoxicity and ensure early intervention​ [48].

Cardiotoxicity from cancer therapy, particularly with TZ in breast cancer patients, significantly impacts CV health, necessitating cardiology involvement for optimal care. In a study of 1,047 patients, cardiology involvement was associated with higher rates of guideline-adherent cardiac monitoring (76.4% vs. 60.1%, p = 0.007) and greater utilization of cardiac biomarkers during therapy (27.8% vs. 13.8%, p = 0.001) [49]. The median follow-up period for the study was 26 months, with an interquartile range of 12-47 months, and the median duration of TZ therapy was 11 months, with an interquartile range of 6-12 months. TZ was associated with a well-established risk of LVEF declines, cardiomyopathy, and HF, with the cardiotoxicity risk being particularly high when used sequentially with ACs, leading to asymptomatic declines in LVEF and symptoms of HF. Treatment with ACs was more frequent among patients with cardiology involvement (18.8% vs. 11.8%), and AC therapy was independently associated with increased cardiology involvement during follow-up, underscoring its significant cardiotoxic potential [49]. Monitoring includes regular echocardiography and biomarker assessments, while management emphasizes adherence to CV guidelines and addressing pre-existing CV risk factors to mitigate cardiotoxic effects [49].

Baseline cardiac imaging is essential in managing cardiotoxicity from cancer therapies, particularly in women with early-stage breast cancer undergoing AC or TZ treatments. In a study of 11,732 women, 79% receiving AC and 81% receiving TZ had baseline cardiac imaging, showing significant adherence to guidelines ​[20]. However, only 16% of those on non-AC regimens received such imaging despite higher cancer stages and worse tumor grades in this group. Improved monitoring and management practices are necessary, particularly for those at higher CV risk, to optimize patient outcomes and guide treatment decisions ​​[20]​.

ICIs, Programmed Cell Death Protein 1, Tyrosine Kinase Inhibitor

Cardiotoxicity from ICIs is a significant concern, particularly with late CAEs occurring after 90 days of treatment initiation. In a cohort of 19 patients with late ICIs-associated CAEs, the median time to onset was 304 days, with 73.7% experiencing LVSD and 47.4% developing HF [53]. The mortality rates were similarly high in both early and late CAE cases (40% vs. 44.4%). Notably, high-dose corticosteroids were commonly used in these patients, and management strategies involved beta blockers, ACE inhibitors, and ivabradine for symptomatic relief and recovery of LV function. Continuous cardiac monitoring beyond the initial 90 days of ICI therapy is recommended to detect and manage these late-onset cardiac complications effectively​ [53]. Among 153 cancer patients under treatment with DOX and ICIs who underwent coronary angiography, 66% presented with acute coronary syndrome (ACS), and the one-year mortality was notably higher at 46% compared to 8% in non-cancer patients. Troponin-positive ACS patients had a five-year mortality rate of 71% [47]. Monitoring involves using cardiac biomarkers such as troponin and brain natriuretic peptides to detect early signs of cardiotoxicity. Management should follow CV guidelines, suggesting dual antiplatelet therapy durations similar to the general population despite the challenges posed by low platelet counts and bleeding risks [47]​.

Cardiotoxicity from ICIs in elderly patients is relatively low but significant, requiring diligent monitoring and management. In a study analyzing the cardiotoxicity associated with ICIs in 195 elderly cancer patients, the median follow-up period was 26 months, with an interquartile range (IQR) of 12-47 months​​ [24]. TZ therapy, a common treatment for breast cancer, had a median duration of 11 months (IQR 6-12 months)​​. TZ is known to pose a significant risk of cardiotoxicity, including declines in LVEF, cardiomyopathy, and HF, particularly when used sequentially with ACs​ [24]​. ACs themselves were also associated with substantial cardiotoxic potential, with a higher frequency of use observed in patients requiring cardiology involvement during follow-up (18.8% vs. 11.8%) [24]. The study highlights the necessity for careful cardiovascular monitoring in patients receiving these treatments due to their notable risks of cardiotoxic events​. In a study involving 500 patients with lung and esophageal cancer treated with ICIs, the mean baseline LVEF was 68.51% ± 4.81%, which significantly decreased to 66.23% ± 4.20% after 12 months (p < 0.001) [28]. Cardiac troponin-T (cTnT) levels increased from a baseline of 7.42 ± 3.95 ng/L to 14.54 ± 14.49 ng/L (p < 0.001). The extracellular volume (ECV) score, indicating myocardial fibrosis, increased from 47.14% ± 7.48% to 52.64% ± 7.58% after 12 months (p < 0.001) [28]. Cardiac dysfunction related to cancer therapy occurred in 9.8% of patients, with significant increases in cTnT and ECV in those affected. Monitoring through contrast-enhanced chest CT and measuring ECV provides early detection of myocardial damage, allowing timely intervention to manage cardiotoxicity effectively​ [28].

Ibrutinib, a tyrosine kinase inhibitor (TKI), is associated with a higher incidence of atrial and ventricular arrhythmias compared to non-Bruton tyrosine kinase (BTK) TKIs and non-TKI therapies in patients with hematologic malignancies. In a study involving 193 patients, AF was observed in 44% of those on ibrutinib, significantly higher than the 15% in the non-BTK TKI group and 20% in the non-TKI group (p = 0.001 and p = 0.002, respectively) [27]. Non-sustained ventricular tachycardia occurred in 43% of the ibrutinib group compared to 17% and 27% in the non-BTK TKI and non-TKI groups, respectively (p = 0.004 and p = 0.04). Due to these arrhythmias, ibrutinib therapy was interrupted in 25% of patients, significantly higher than the 4% interruption rate in the non-BTK TKI group (p = 0.005). Monitoring and managing these arrhythmias necessitate regular CV assessments and the involvement of cardiologists to optimize treatment strategies and mitigate the risk of serious cardiac events​ [27]​.

In a cohort study of 33 patients treated with ibrutinib, nearly two-thirds exhibited signs of myocardial damage, with over 50% having LGE fibrosis. The study revealed that post-treatment native T1 values increased significantly from 977.0 ms to 1033.7 ms, and max-T2 values rose from 56.5 ms to 61.5 ms [35]. Furthermore, 54.8% of patients developed LGE post-treatment compared to 13.3% pre-treatment (p < 0.001). Over a median follow-up of 19 months, 39.4% of patients experienced major adverse cardiac events. Monitoring and management strategies emphasize the use of CMR for early detection of subclinical myocardial injury and the need for comprehensive cardiac monitoring, including regular ECGs and biomarker assessments, to manage and mitigate cardiotoxic risks effectively ​[35].

The study involving 24,129 patients with NSCLC treated with TKI found an increased incidence of major adverse cardiac and cerebrovascular events (MACCEs), including HF (adjusted subdistribution HR, 1.10; 95% CI, 1.02-1.19), acute myocardial infarction (adjusted subdistribution HR, 1.27; 95% CI, 1.06-1.51), and ischemic stroke (adjusted subdistribution HR, 1.34; 95% CI, 1.24-1.44) compared to those not treated with TKIs ​[38]. Despite the increased risk of MACCEs, TKI use was associated with a lower risk of all-cause death (adjusted HR, 0.76; 95% CI, 0.75-0.78). Management strategies include regular CV monitoring and early intervention with medications such as ACE inhibitors and beta-blockers to mitigate cardiotoxic effects and optimize patient outcomes ​[38].

Fluoropyrimidines

Fluoropyrimidines (FP) in colorectal cancer patients have been reported to present significant risks. In a prospective study involving 129 patients, 15.5% experienced FP-induced cardiotoxicity, with common symptoms including dyspnea (60%), chest pain (40%), and palpitations (40%) ​[34]. Notably, 15% had clinically relevant ECG changes, including one acute myocardial infarction. Despite 48% having pre-existing cardiac conditions, increased cardiotoxicity was not observed in this subgroup, although females with high alcohol consumption showed higher susceptibility. Monitoring and management strategies include baseline cardiological assessments, regular ECG and biomarker monitoring, and the use of cardiological protective therapies when necessary. With careful monitoring, nearly all patients completed their FP treatment without recurrence of cardiotoxicity ​[34].

Cancer therapy with 5-fluorouracil (5-FU)-based regimens also poses a significant concern, with incidence rates of symptomatic cardiotoxicity ranging from 1% to 20% [32]. Studies indicate that over 50% of patients on 5-FU may experience asymptomatic ST segment alterations indicative of decreased myocardial perfusion. The primary mechanism behind 5-FU-induced cardiotoxicity is believed to be microvascular dysfunction, specifically involving the endothelial nitric oxide synthase pathway. Patients treated with 5-FU showed significantly impaired cutaneous microvascular reactivity, with reduced %ΔCVC (566 ± 305% in controls vs. 282 ± 172% in 5-FU patients, p = 0.001), highlighting the impact on endothelial function [32]. Management and monitoring of cardiotoxicity include regular CV assessments, patient education on symptom reporting, and potential use of cardioprotective agents such as beta-blockers and ACE inhibitors. Multidisciplinary approaches and individualized treatment plans are recommended to mitigate the CV risks associated with 5-FU therapy​ [32]​.

Oncolytics

In a study of 67 patients who received oral oncolytics (aromatase inhibitors (36%), breakpoint cluster region-Abelson murine leukemia inhibitors (16%), and vascular endothelial growth factor receptor inhibitors (13%), 46.2% experienced HF with a median onset of 148 days [29]. The median follow-up period was 26 months, and the duration of TZ therapy was a median of 11 months. The study highlighted significant cardiotoxic risks associated with TZ and ACs. TZ was linked to declines in LVEF, cardiomyopathy, and HF, with increased cardiotoxicity risk when used sequentially with ACs, causing both asymptomatic LVEF declines and symptomatic HF [29]. ACs were more frequently used in patients with cardiology involvement and were independently associated with increased cardiology involvement during follow-up, indicating their significant cardiotoxic potential [29]. Cardioprotective interventions included pharmacological treatment in 65.7% of cases, treatment interruption in 26.9%, and dose reductions in 10.4% [29]. Elevated troponins were observed in 45.2% of HF patients, and B-type natriuretic peptide (BNP) was elevated in 77.4% at presentation [29]. Monitoring strategies emphasized the use of echocardiograms and biomarkers such as troponins and BNP for early detection [29]. Multidisciplinary approaches and adherence to guideline-directed medical therapy are crucial for managing cardiotoxic events and deciding whether to continue or discontinue cancer therapy​ [29]​.

Biomarkers

Cardiotoxicity from cancer therapy, particularly with AC, can be predicted using AI models based on baseline ECG. In a study of 1,011 AC-treated patients, 8.7% developed CTRCD, with high AI-CTRCD scores indicating a significantly elevated risk (HR 2.66; 95% CI, 1.73-4.10). The AI-CTRCD score demonstrated superior predictive power, enhancing the AUC from 0.74 to 0.78 for two-year CTRCD prediction [13]​. Patients with high scores had an incidence rate of 8.93 per 100 person-years, compared to 3.08 in low-score patients. Monitoring strategies should include baseline ECG enhanced with AI-CTRCD models to identify high-risk patients, facilitating timely echocardiograms and cardioprotective interventions ​[13]​. In a study of 32 breast cancer patients, GLS worsened significantly from baseline (-19.1 ± 2.1%) to three months (-16.0 ± 3.1%) and six months (-16.1 ± 3.0%) post-therapy (p < 0.001). Univariable Cox regression showed that a 1-SD increase in GLS was associated with a 2.1-fold increased risk of CTRCD (HR 2.1; 95% CI, 1.4-3.1; p < 0.001). This finding was independent of LVEF, which remained unchanged. For monitoring and management, the study highlights the importance of early GLS assessment and regular follow-ups to predict and mitigate long-term cardiotoxicity risks, thus enabling timely interventions such as cardioprotective therapies ​[14].

Cardiotoxicity from cancer therapy can be diagnosed and managed using early biomarkers and imaging techniques. A study involving breast cancer patients treated with AC and sequential targeted therapy found that GLS at three months post-therapy was a strong predictor of late-onset CTRCD, with an AUC of 0.745 and a cut-off value of 20.32% [12]​. Additionally, the combination of GLS and contrast-enhanced LVEF at three months provided an even higher predictive value for CTRCD (AUC = 0.929). Over a two-year follow-up, the incidence of CTRCD was 30.6%, with significant declines in GLS and LVEF and increases in the E/e' ratio observed particularly in patients receiving combined AC and targeted therapy. Monitoring these parameters early and regularly can help in timely interventions to adjust cancer treatment and minimize cardiac damage​ [12].

In an international, multicenter, randomized controlled trial (SUCCOUR study) with 331 AC-treated patients, the use of GLS to guide CPT significantly reduced the incidence of CTRCD compared to an EF-guided approach. Specifically, 5.8% of patients in the GLS-guided arm developed CTRCD compared to 13.7% in the EF-guided arm (p = 0.02). Additionally, patients in the GLS-guided arm who received CPT had a significantly lower reduction in LVEF at the one-year follow-up compared to those in the EF-guided arm (2.9% vs. 9.1%; p = 0.03) [44]. Monitoring involved baseline and regular echocardiographic assessments every three months, focusing on GLS and LVEF to detect subclinical myocardial dysfunction early. This strategy allowed for the timely initiation of ACE inhibitors and beta-blockers, which are crucial for mitigating severe cardiac dysfunction and maintaining optimal cardiac function during and after cancer therapy [44].

Two-dimensional (2D) STE is more sensitive and precise than traditional echocardiography in detecting early LV dysfunction caused by AC in breast cancer patients. In a study of 33 patients, conventional echocardiographic parameters did not show significant changes after four cycles of chemotherapy, except for a slight reduction in LVEF (64.00% at T0 to 63.42% at T4, p < 0.05) [23]. However, 2D-STE parameters, such as LVGLS, showed significant deterioration after just two cycles; LVGLS decreased from -21.34% at T0 to -19.85% at T2, p < 0.01) [23]. This suggests that 2D-STE can detect myocardial damage earlier, allowing for timely intervention. For monitoring and managing cardiotoxicity, regular use of 2D-STE is recommended to identify subclinical changes and implement cardioprotective strategies before significant damage occurs​ [23].

Patients treated with AC, alkylating agents, taxanes, and programmed cell death protein 1 inhibitors frequently exhibited elevated levels of NT-proBNP, with 43% of those with LVEF below 50% showing no NT-proBNP increase​ ​[52]​​. Monitoring and managing cardiotoxicity involves early identification of myocardial dysfunction, crucial for modifying treatment to less cardiotoxic options and initiating cardioprotective strategies. Biomarkers such as NT-proBNP and cardiac troponin I are valuable for detection, although their low sensitivity necessitates complementary methods such as anamnesis, clinical examinations, electrocardiography, and echocardiography for a comprehensive assessment ​[52]​.

Cardiotoxicity from cancer therapy significantly affects LV systolic function, necessitating early detection and monitoring strategies. In a study of 30 cancer patients undergoing chemotherapy and/or radiotherapy, STE detected significant reductions in GLS from -21.16 ± 2.50 to -19.86 ± 3.22 (p < 0.01) and global circumferential strain from -23.60 ± 7.36 to -21.33 ± 6.97 (p < 0.01) over six months, despite preserved LVEF [50]. This indicates that STE is more sensitive in identifying early myocardial dysfunction than traditional 2D echocardiography. For monitoring and management, regular STE assessments are recommended to detect subclinical cardiotoxicity early, allowing timely intervention with cardioprotective agents such as dexrazoxane to prevent irreversible cardiac damage​ [50].

Cardiotoxicity from cancer therapy, particularly involving AC and TZ, leads to significant changes in LV function parameters, emphasizing the need for robust monitoring. In a study with 30 breast cancer patients, those who developed CTRCD exhibited larger temporal changes in LV function. The mean temporal change in 2D echocardiographic GLS was 1.9% in patients with CTRCD compared to 0.7% in those without. Similarly, CMR-LVEF changes were 6.6% in patients with CTRCD compared to 2.7% in those without [51]​. The best measures to differentiate CTRCD included echocardiographic 3D-LVEF, 2D-GLS, and CMR-LVEF. For effective monitoring, regular cardiac imaging using echocardiography or CMR is recommended to detect subclinical changes early. Management involves close monitoring of cardiac function and adherence to guidelines for timely intervention with cardioprotective strategies​ [51]​.

## Conclusions

Cardiotoxicity resulting from cancer therapy, particularly CRT for NSCLC and treatments involving ACc and TZ for breast cancer, is a significant complication that negatively impacts patient survival and quality of life. Biomarkers such as high-sensitivity cardiac troponin T and N-terminal pro-B-type natriuretic peptide have proven effective in the early detection of cardiotoxicity. Additionally, advanced imaging techniques such as positron emission tomography/computed tomography and cardiovascular magnetic resonance imaging are crucial for monitoring and managing cardiac dysfunction. Regular monitoring of biomarkers and echocardiographic parameters, such as LVEF and GLS, enables early identification of high-risk patients, allowing timely interventions and therapy modifications to mitigate cardiotoxicity. A proactive approach involving close collaboration between oncologists and cardiologists is essential for improving patient outcomes by reducing cardiovascular risks and enhancing quality of life.

Therefore, early detection and effective management of cardiotoxicity are vital in cancer therapy. Utilizing biomarkers, advanced imaging techniques, and implementing personalized cardioprotective strategies are crucial measures to minimize adverse cardiac effects and ensure safer and more effective cancer treatment.

## Appendices

**Table 3:** Analysis of study quality/bias Y = yes, N = no, NC = not clear, N/A = not applicable

Checklist Question	Xu et al. [9]	Kim et al. [10]	Demissei et al. [11]	Liu et al. [12]	Yagi et al. [13]	Kar et al. [14]	Upshaw et al. [15]	Jacobs et al. [16]	Koop et al. [17]	Kinoshita et al. [18]	Chang et al. [19]	Barac et al. [20]	Kersten et al. [21]	Sang et al. [22]	Chen et al. [23]	​Toribio-García et al. [24]	Shibata et al. [25]	Glen et al. [26]	Wei et al. [27]	Wang et al. [28]	Abboud et al. [29]	Gąsior et al. [30]	Di Lisi et al. [31]	Hammond et al. [32]	Posch et al. [33]	Lombardi et al. [34]	Buck et al. [35]​	Lu et al. [36]	Valente et al. [37]	Chang et al. [38]	Beukema et al. [39]	Hu et al. [40]	Lee et al. [41]	Carpenter et al. [42]	Saijo et al. [43]	Thavendiranathan et al. [44]	Laufer-perl et al. [45]	​Esteban-Fernández et al. [46]	Mrotzek et al. [47]	Micheletti et al. [48]	​Demissei et al. [49]	Singh et al. [50]	Lambert et al. [51]	Hinrichs et al. [52]	Dolladille et al. [53]
Were there clear criteria for inclusion in the case series?	Y	Y	Y	Y	Y	Y	Y	Y	Y	Y	Y	Y	Y	Y	Y	Y	Y	Y	Y	Y	Y	Y	Y	Y	Y	Y	Y	Y	Y	Y	Y	Y	Y	Y	Y	Y	Y	Y	Y	Y	Y	Y	Y	Y	Y
Was the condition measured in a standard, reliable way for all participants included in the case series?	Y	Y	Y	Y	Y	Y	Y	Y	Y	Y	Y	Y	Y	Y	Y	Y	Y	Y	Y	Y	Y	Y	Y	Y	Y	Y	Y	Y	Y	Y	Y	Y	Y	Y	Y	Y	Y	Y	Y	Y	Y	Y	Y	Y	Y
Were valid methods used for the identification of the condition for all participants included in the case series?	Y	Y	Y	Y	Y	Y	Y	Y	Y	Y	Y	Y	Y	Y	Y	Y	Y	Y	Y	Y	Y	Y	Y	Y	Y	Y	Y	Y	Y	Y	Y	Y	Y	Y	Y	Y	Y	Y	Y	Y	Y	Y	Y	Y	Y
Did the case series have the consecutive inclusion of participants?	Y	Y	Y	Y	N	N	Y	Y	N/A	NC	Y	NC	Y	Y	Y	Y	Y	Y	Y	Y	Y	Y	Y	Y	Y	Y	Y	Y	NC	Y	Y	Y	Y	Y	Y	NC	Y	Y	NC	NC	NC	NC	NC	NC	Y
Did the case series have a complete inclusion of participants?	Y	Y	Y	Y	N	N	Y	N	N	Y	Y	N	Y	Y	Y	Y	Y	N	Y	Y	Y	Y	Y	Y	Y	N	Y	Y	Y	Y	Y	Y	N	N	Y	N	N	Y	Y	Y	Y	Y	Y	Y	Y
Was there clear reporting of the demographics of the participants in the study?	Y	Y	Y	Y	Y	Y	Y	Y	Y	Y	Y	Y	Y	Y	Y	Y	Y	Y	Y	Y	Y	Y	Y	Y	Y	Y	Y	Y	Y	Y	Y	Y	Y	Y	Y	Y	Y	Y	Y	Y	Y	Y	Y	Y	Y
Were the outcomes or follow-up results of cases clearly reported?	Y	Y	Y	Y	Y	Y	Y	Y	Y	Y	Y	Y	Y	Y	Y	Y	Y	Y	Y	Y	Y	Y	Y	Y	Y	Y	Y	Y	Y	Y	Y	Y	Y	Y	Y	Y	Y	Y	Y	Y	Y	Y	Y	Y	Y
Was there clear reporting of the presenting sites’ or clinics’ demographic information?	Y	Y	Y	Y	Y	Y	Y	Y	Y	Y	Y	Y	Y	Y	Y	Y	Y	Y	Y	Y	Y	Y	Y	Y	Y	Y	Y	Y	Y	Y	Y	Y	Y	Y	Y	Y	Y	Y	Y	Y	Y	Y	Y	Y	Y
Was the statistical analysis adequate?	Y	Y	Y	Y	Y	Y	Y	Y	Y	N	Y	Y	Y		Y	Y	Y	Y	Y	Y	Y	Y	Y	Y	Y	Y	Y	Y	Y	Y	Y	Y	Y	Y	Y	Y	Y	Y	Y	Y	Y	Y	Y	Y	Y
